# Abiraterone-Induced Hypokalemia and Hypophosphatemia: A Case Report

**DOI:** 10.7759/cureus.110321

**Published:** 2026-06-05

**Authors:** Adnan Safi, Ayesha Fayyaz, Fnu Veerban, Syed Jahangir, Hari Vishal, Emmanuel Akatibo, Gnama Kouyate, Mary Mallappallil, Isha Puri, Ibrahim Mohamed, Muhammad Azhar

**Affiliations:** 1 Nephrology, State University of New York Downstate Health Sciences University/ Kings County Hospital Center, Brooklyn, USA; 2 Internal Medicine, State University of New York Downstate Health Sciences University/ Kings County Hospital Center, Brooklyn, USA; 3 Medicine, State University of New York Downstate Health Sciences University/ Kings County Hospital Center, Brooklyn, USA

**Keywords:** abiraterone acetate, hypophosphatemia, mineralocorticoid excess, prostate cancer, refractory hypokalemia, spironolactone

## Abstract

Abiraterone acetate is a commonly used androgen biosynthesis inhibitor in the management of advanced prostate cancer and is associated with mineralocorticoid excess due to CYP17 inhibition. This can result in electrolyte abnormalities, most notably hypokalemia, which may be severe and refractory to standard replacement therapy. We present the case of an 82-year-old male with metastatic prostate cancer on abiraterone therapy who was admitted with altered mental status, hyponatremia, and acute kidney injury in the setting of poor oral intake. Despite appropriate volume resuscitation and electrolyte replacement, the patient developed persistent hypokalemia and hypophosphatemia with evidence of renal potassium wasting. Given the clinical context, abiraterone-induced mineralocorticoid excess was suspected. As discontinuation of abiraterone was not feasible, treatment with the mineralocorticoid receptor antagonist spironolactone was initiated, resulting in sustained correction of electrolyte abnormalities. This case highlights the need to consider abiraterone-induced mineralocorticoid excess in patients with refractory hypokalemia, particularly when renal potassium wasting persists after correction of volume status and kidney function. Early recognition may prevent prolonged hospitalization, treatment delays, and serious complications. When abiraterone must be continued, mineralocorticoid receptor antagonists can provide targeted therapy while preserving cancer treatment. This case also emphasizes the importance of routine electrolyte monitoring in patients receiving abiraterone, even when they are prescribed concurrent prednisone.

## Introduction

Prostate cancer is one of the most common malignancies affecting men and frequently requires long-term androgen deprivation therapy in advanced stages. Abiraterone acetate is a selective and irreversible inhibitor of cytochrome P450 17α-hydroxylase (CYP17) and is widely used in the treatment of metastatic prostate cancer due to its demonstrated survival benefit in both castration-resistant and castration-sensitive disease [1,2]. By inhibiting CYP17, abiraterone suppresses androgen and cortisol synthesis in the adrenal glands and tumor tissue. Reduced cortisol levels lead to loss of negative feedback on adrenocorticotropic hormone (ACTH), resulting in increased production of mineralocorticoid precursors such as deoxycorticosterone. This mechanism can cause a syndrome of mineralocorticoid excess, clinically manifested by hypertension, fluid retention, and hypokalemia [3]. These adverse effects are well described and may occur despite routine co-administration of low-dose glucocorticoids [4].

Abiraterone-associated hypokalemia is not rare, but severe or refractory presentations are less common. In the COU-AA-302 trial, hypokalemia of any grade occurred in 17% of patients receiving abiraterone plus prednisone, while grade 3-4 hypokalemia occurred in 2.6% of patients [1]. In the LATITUDE trial, grade 3-4 hypokalemia occurred in approximately 10% of patients receiving abiraterone compared with 1% in the placebo group [2]. Persistent hypokalemia with renal potassium wasting and associated hypophosphatemia has been infrequently reported in the literature [5,6].

Abiraterone-associated hypokalemia is a recognized adverse effect and may be severe or persistent, leading to significant morbidity, including cardiac arrhythmias, seizures, and neuromuscular weakness [5]. Awareness of this complication is essential, particularly when electrolyte abnormalities are refractory to standard replacement therapy. We present a case of abiraterone-induced mineralocorticoid excess with persistent hypokalemia and hypophosphatemia that required targeted therapy with a mineralocorticoid receptor antagonist.

## Case presentation

An 82-year-old man with metastatic prostate cancer diagnosed in March 2024 involving the left common iliac chain lymph node, previously treated with external beam radiation therapy, and currently receiving abiraterone acetate 1000 mg daily, prednisone 5 mg daily, and leuprolide 45 mg every six months, presented to the emergency department with poor oral intake for three days and progressive altered mental status. His medical history was also significant for type 2 diabetes mellitus, hypertension, chronic kidney disease stage 2, and a prior right-sided cerebrovascular accident.

According to his family, the patient had a possible seizure-like episode at home prior to presentation. There was no history of fever, chills, rigors, or recent infection. For several days before admission, he had poor oral intake associated with nausea and vomiting.

On arrival, the patient was mildly confused and appeared dehydrated and hypovolemic. Vital signs showed a blood pressure of 105/61 mmHg, heart rate of 81 beats per minute, respiratory rate of 19 breaths per minute, and temperature of 98°F. Physical examination was otherwise unremarkable, with no focal neurological deficits.

Initial laboratory evaluation revealed significant hyponatremia with serum sodium of 120 mEq/L (reference range: 135-145 mEq/L), hypokalemia with serum potassium of 3.0 mEq/L (reference range: 3.5-5.0 mEq/L), hypochloremia with serum chloride of 85 mEq/L (reference range: 98-106 mEq/L), and acute kidney injury with serum creatinine of 1.9 mg/dL (reference range: 0.5-0.9 mg/dL; baseline 1.5 mg/dL) and blood urea nitrogen of 74 mg/dL (reference range: 8-23 mg/dL). Additional findings included anemia with hemoglobin of 8.6 g/dL (reference range: 12-16 g/dL), mild eosinophilia with eosinophil count of 11% (reference range: 0-8.6%), and hypoalbuminemia with albumin of 3.0 g/dL (reference range: 2.8-5.7 g/dL). Initial phosphorus was elevated at 5.6 mg/dL in the setting of acute kidney injury but subsequently declined to 1.9 mg/dL as renal function improved, revealing persistent hypophosphatemia. Urine studies demonstrated inappropriately elevated urinary potassium levels ranging from 41-54 mEq/L, supporting ongoing renal potassium wasting. Urine sodium was 84 mEq/L, and urine osmolality was 222 mOsm/kg.

The patient was initially diagnosed with hypovolemic hyponatremia and prerenal acute kidney injury in the setting of poor oral intake and vomiting. He was started on isotonic intravenous fluids with careful monitoring, targeting a gradual sodium correction of no more than 10 mEq/L in 24 hours. His serum sodium levels improved appropriately, accompanied by a gradual improvement in mental status.

Although the urine studies raised consideration of SIADH and adrenal insufficiency, the history of vomiting and poor oral intake, clinical hypovolemia, low urine chloride, and prompt response to isotonic fluids favored hypovolemic hyponatremia.

Renal function initially showed limited improvement, raising concern for mild acute tubular necrosis superimposed on pre-renal injury. Over the following days, his serum creatinine progressively improved and returned close to baseline. Despite recovery of renal function and adequate electrolyte replacement, the patient continued to have persistent hypokalemia and hypophosphatemia and ongoing abiraterone therapy; medication-induced mineralocorticoid excess was considered the most likely explanation. Abiraterone could not be discontinued due to oncologic considerations.

Adrenal insufficiency and adrenal crisis were considered in the differential diagnosis because the patient presented with vomiting, altered mental status, hyponatremia, and acute kidney injury. However, adrenal crisis was considered less likely because the patient did not exhibit refractory hypotension, severe hypoglycemia, or hyperkalemia. Instead, he demonstrated persistent hypokalemia with elevated urinary potassium excretion, suggesting renal potassium wasting. Additionally, his clinical status improved with intravenous fluid resuscitation without the need for stress-dose glucocorticoids. These findings were more consistent with abiraterone-induced mineralocorticoid excess than adrenal insufficiency [3,4].

To counteract the mineralocorticoid effects of abiraterone, spironolactone was initiated. Following initiation, the patient’s serum potassium and phosphorus levels showed sustained improvement, further supporting the diagnosis of abiraterone-induced mineralocorticoid excess.

## Discussion

Abiraterone disrupts adrenal steroidogenesis through inhibition of CYP17, resulting in decreased cortisol synthesis and compensatory elevation of ACTH. The subsequent accumulation of mineralocorticoid intermediates, particularly deoxycorticosterone, produces aldosterone-like effects at the renal distal tubules, leading to sodium retention and renal potassium wasting [3]. This mechanism likely explains why some patients remain hypokalemic despite aggressive potassium replacement.

Several case reports and clinical studies have demonstrated that abiraterone-induced hypokalemia is frequently renal in origin and may be resistant to electrolyte replacement alone. Elevated urinary potassium excretion supports ongoing renal losses rather than reduced intake or intracellular shifts [5,6]. Our patient exhibited similar findings, with persistent hypokalemia despite correction of volume status and aggressive supplementation.

Although adrenal insufficiency may be considered in patients receiving abiraterone because of suppression of cortisol synthesis, the clinical presentation in this case was more consistent with mineralocorticoid excess. The patient did not develop refractory hypotension, severe hypoglycemia, or hyperkalemia, which are more typical features of adrenal crisis. Instead, persistent renal potassium wasting with elevated urinary potassium excretion supported ongoing mineralocorticoid activity as the primary mechanism [3,4].

A limitation of this report is that serum ACTH, cortisol concentrations, and urine steroid profiling were not obtained during hospitalization. Although these investigations may have provided additional biochemical confirmation of mineralocorticoid excess, the diagnosis was supported by the patient's exposure to abiraterone, persistent renal potassium wasting despite correction of volume status, elevated urinary potassium excretion, exclusion of more likely alternative etiologies, and sustained improvement following initiation of spironolactone.

Using the Naranjo Adverse Drug Reaction Probability Scale, this case yielded an estimated score of 6, suggesting a probable adverse drug reaction related to abiraterone therapy [7]. The score was supported by prior reports of abiraterone-associated hypokalemia, the temporal relationship with therapy, persistent renal potassium wasting, and improvement after spironolactone initiation.

Although low-dose prednisone is routinely prescribed with abiraterone to suppress ACTH secretion, mineralocorticoid excess may still occur. Clinical trial data indicate that hypokalemia occurs in a significant proportion of patients receiving abiraterone, with severe cases reported in a minority [1, 2]. In rare instances, untreated electrolyte disturbances have resulted in life-threatening complications, including ventricular arrhythmias and cardiac arrest [8].

Eplerenone may also be considered because of its lower antiandrogenic activity; however, spironolactone was selected in this case because of availability, severity of the electrolyte abnormalities, and favorable clinical response. Although spironolactone possesses partial androgen receptor activity and has raised theoretical concerns regarding prostate cancer progression, available evidence remains limited, and the decision was individualized based on the need for rapid correction of mineralocorticoid-mediated electrolyte disturbances while maintaining continuity of oncologic therapy.

This approach allows continuation of abiraterone therapy while addressing the underlying mechanism of electrolyte imbalance. Modification of oncologic therapy was considered; however, continuation of abiraterone was favored because of ongoing oncologic benefit and the patient’s established treatment response. Therefore, management focused on correcting the underlying mineralocorticoid excess while maintaining continuity of cancer therapy. In our patient, initiation of spironolactone resulted in sustained improvement of potassium and phosphorus levels, supporting this management strategy.

## Conclusions

This case highlights abiraterone-induced mineralocorticoid excess as an important cause of refractory hypokalemia and renal potassium wasting in patients with advanced prostate cancer. Although adrenal insufficiency may be considered in patients receiving abiraterone, the presence of persistent hypokalemia, elevated urinary potassium excretion, absence of refractory hypotension or hyperkalemia, and response to spironolactone favored mineralocorticoid excess rather than adrenal crisis. Severe hypokalemia occurs in a minority of patients receiving abiraterone, but persistent renal potassium wasting with associated hypophosphatemia remains relatively uncommon. Early recognition and treatment with mineralocorticoid receptor antagonists may help correct electrolyte abnormalities while allowing continuation of cancer therapy.
